# Eating in the absence of hunger is not associated with weight, self-reported eating behaviors, or well-being in pregnant adults: Prospective cohort study

**DOI:** 10.1371/journal.pone.0325478

**Published:** 2025-06-24

**Authors:** Leah M. Lipsky, Kyle S. Burger, Myles S. Faith, Grace E. Shearrer, Tonja R. Nansel

**Affiliations:** 1 Social and Behavioral Sciences Branch, Eunice Kennedy Shriver National Institute for Child health and Human Development, National Institutes of Health, Bethesda, Maryland, United States of America; 2 Department of Nutrition, Gillings School of Global Public Health, University of North Carolina, Chapel Hill, North Carolina, United States of America; 3 Department of Counseling, School and Educational Psychology, University at Buffalo Graduate School of Education, Buffalo, New York, United States of America; 4 Department of Family and Consumer Sciences, University of Wyoming, Laramie, Wyoming, United States of America; Pennington Biomedical Research Center, UNITED STATES OF AMERICA

## Abstract

**Objective:**

Eating in the absence of hunger (EAH) refers to consuming food after reaching satiation and is considered a risk factor for weight gain. This study examined relations of EAH with pregnancy-related weight outcomes, self-reported eating behaviors, and indicators of well-being.

**Methods:**

EAH was measured in participants (n = 46) during their 2^nd^ pregnancy trimester. Energy intake and percent of food consumed following a standardized meal was calculated for all foods, and separately for sweet (desserts) and savory (salty snacks) foods. Early pregnancy BMI, gestational weight gain, and postpartum weight change were calculated from measured height and weight from <12 weeks gestation to 1 year postpartum. Participants reported eating behaviors (Dutch Eating Behavior Questionnaire), depressive symptoms (Edinburgh Postnatal Depression Scale), stress (Perceived Stress Scale), and sleep quality (Pittsburgh Sleep Quality Index) < 28 weeks gestation. Linear and logistic regression models estimated relationships between the variables of interest.

**Results:**

Primarily null estimates did not provide consistent evidence of associations of eating behaviors or indicators of well-being with EAH, or of EAH with pregnancy-related weight outcomes.

**Conclusions:**

EAH in pregnancy was not related to weight change, eating behaviors, depressive symptoms, sleep quality, or stress. Future studies in larger samples and diverse developmental periods are needed to determine the utility of laboratory-assessed EAH as a risk factor for weight gain.

**Trial registration:**

ClinicalTrials.gov NCT02217462

## Introduction

High pre-pregnancy BMI and excessive gestational weight gain (GWG) impact a majority of pregnancies in the U.S. [1], increasing risk for postpartum weight retention [2] and adverse health outcomes for the childbearing parent and infant [3]. Excessive GWG is primarily attributable to maternal fat accumulation [4] and likely due to excessive energy intake relative to expenditure [5]. However, interventions to optimize pregnancy weight outcomes have yielded inconsistent effects [6]. Better understanding of behavioral correlates of suboptimal pregnancy weight outcomes is needed to inform intervention development during this vulnerable life stage.

Eating in the absence of hunger refers to eating beyond perceived physiological hunger and is hypothesized to contribute to obesity development [7]. EAH can be measured under controlled conditions by quantifying participants’ *ad libitum* intake of snack foods provided after having consumed a meal to self-reported satiation (i.e., fullness or absence of hunger). Most research on EAH has been conducted in children to investigate correlates of overweight and to identify genetic factors and parent behaviors contributing to child EAH. A recent systematic review reported that findings from 31 cross-sectional studies of the relationship of EAH with adiposity indicators in children (typically BMI percentile or z-score) have been mixed, including both positive and null relationships [8]; while the five prospective studies reported positive associations of EAH with adiposity, four of these were from the same cohort, and most examined EAH as an outcome of high baseline BMI, rather than weight gain as an outcome of EAH. Findings from three prospective studies in children [9–11] and one prospective study in adults [7] examining associations of EAH with prospective weight change have been inconsistent. Other than our previous work [12,13], no previous studies have examined EAH in pregnant samples. Additionally, the relationship of EAH with pregnancy-related weight change has not been examined. More prospective data are needed to determine whether EAH is a behavioral risk factor for adverse weight outcomes during pregnancy and postpartum, as eating behaviors and risk factors for pregnancy-related weight change may differ from those in other developmental stages.

In non-pregnant individuals, self-reported eating behaviors, including emotional eating (i.e., eating in response to emotions), external eating (i.e., eating in response to external cues), and restrained eating (i.e., restricting intake to control weight), as measured using the Dutch Eating Behavior Questionnaire, are hypothesized to increase susceptibility to eating beyond energetic needs [14]. However, evidence in adults and adolescents is equivocal, indicating both null and positive associations [15–17]. Some findings suggest that dietary restraint decreases during pregnancy, and that individuals with high restraint prior to pregnancy experience greater food intake during pregnancy [18]. Evidence on the association of restraint with pregnancy weight outcomes is mixed, suggesting that greater dietary restraint is associated with greater GWG [18,19], but lower postpartum weight retention; however, several studies have reported null associations of restraint with pregnancy weight outcomes [19]. While emotional and external eating during pregnancy have been positively associated with GWG in relatively fewer studies [19], evidence on their associations with food intake during pregnancy is lacking. Stress, depression, and poor sleep quality have also been identified as risk factors for excessive food intake, particularly in females [20]. Several studies in controlled settings, mostly in children or adolescents, indicate that poor mental health and sleep quality are related to greater food intake, particularly those high in fat, carbohydrates, and sodium [21–25]. Understanding how these factors relate to EAH during pregnancy is important given that stress, depression, and sleep quality can be more frequent and severe during this period [26].

Elucidating risk factors for EAH during pregnancy and whether EAH is a risk factor for pregnancy-related weight outcomes may help identify susceptible individuals who may benefit from additional support. The purpose of this study was to examine relationships of eating behaviors, mental health, and sleep with EAH during pregnancy, and to investigate relationships of EAH with pregnancy and postpartum weight indicators. We hypothesized that early pregnancy BMI, more external and emotional eating, worse sleep quality, and more depressive symptoms would predict greater EAH. We did not prespecify the direction of association of restrained eating with EAH. We also hypothesized that EAH would predict greater GWG, greater postpartum weight retention, and lower odds of returning to early pregnancy weight.

## Materials and methods

**Fig 1:**
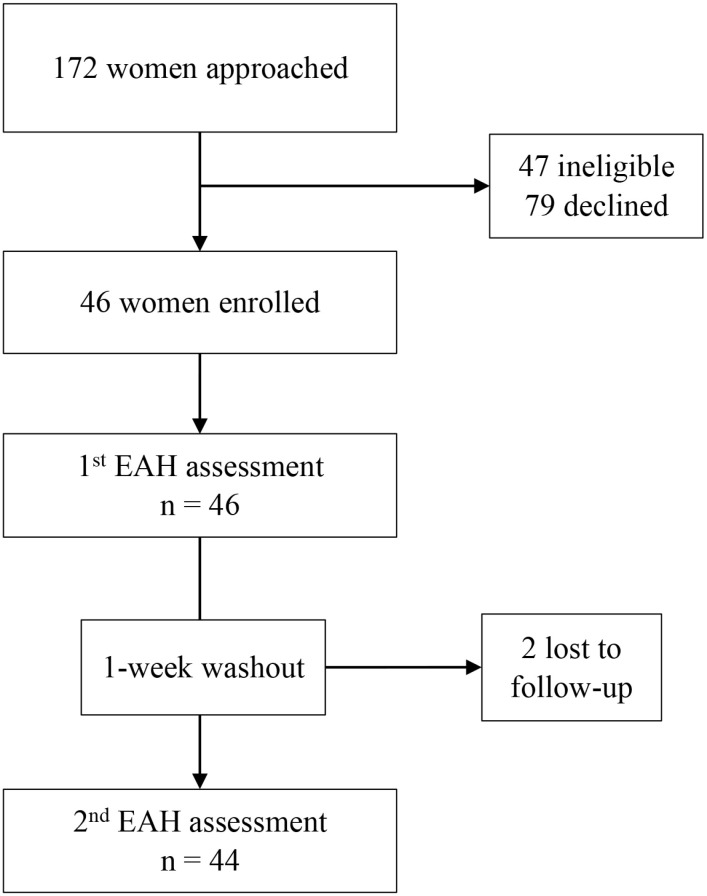
Participant flow diagram.

This is a secondary analysis of data from an EAH substudy within the Pregnancy Eating Attributes Study (PEAS; clinicalTrials.gov identifier: NCT02217462), an observational prospective cohort study of participants followed from early pregnancy (≤12 weeks’ gestation) through 1 year postpartum [27]. Assessments for the main study occurred in each pregnancy trimester and postpartum at 6 weeks, 6 months, and 12 months. The EAH substudy occurred when participants were in their 2^nd^ pregnancy trimester; the primary aim was to test whether EAH in pregnancy differed depending on whether free access foods were highly or minimally processed [13]. For this analysis, only data from the highly processed condition were included in the main analysis, consistent with the hypothesized relationships of EAH with weight outcomes, eating behaviors, and well-being; findings based on the minimally processed condition are provided in the supplemental tables. PEAS was conducted from November 2014 – June 2018; the EAH assessments occurred from November 2015 – September 2016. Eligibility criteria for PEAS included an uncomplicated singleton pregnancy, age ≥ 18 and < 45 years at screening, BMI ≥ 18.5 (calculated from measured height and weight at enrollment), the ability to complete assessments in English, internet and email access, and planning to deliver at The University of North Carolina (UNC) Women’s Hospital and to reside in the vicinity of the clinical site for 1 year postpartum. Exclusion criteria included pre-existing diabetes, multiple pregnancy, participant-reported eating disorder, and any medical or psychological condition or medications affecting diet or weight. Eligible participants for the EAH substudy were further limited to those in their second trimester of pregnancy with active participation in PEAS (completion of at least 75% of first trimester surveys), and prior agreement to consume the foods served in the protocol. The target sample size (n = 50) was selected based on similar EAH studies in adults. Additional details are provided elsewhere [13]. Of the 172 women approached, 47 were ineligible, 79 declined, and 46 were enrolled (Fig 1). EAH participants did not differ from nonparticipants within PEAS with respect to age, BMI category, income-poverty ratio, or race/ethnicity [13]. All participants provided written informed consent. Participants received $20 remuneration after completing the first EAH assessment and $30 remuneration after completing the second EAH assessment. Procedures were approved by the University of North Carolina at Chapel Hill Institutional Review Board.

### EAH

Additional details regarding the EAH procedures have been described previously [13,12]. Briefly, participants completed two separate EAH assessments on two different days at least 1 week apart, each between the hours of 18:00–19:30. Participants rated pre-meal subjective feelings of hunger and fullness and were then provided a standardized meal for *ad libitum* consumption consisting of a pasta dish (cheese tortellini), Caesar salad, and a breadstick (1190 kcal/1650g, 54% carbohydrate, 13% protein, 32% fat). Participants were prompted to eat until they were “no longer hungry.” After the meal, participants rated post-meal hunger and fullness to confirm satiation [13]. After a 10-minute break, the participants then completed a taste test and *ad libitum* snack intake of highly processed (chocolate chip cookies, brownies, Reese’s peanut butter cups, Ruffles potato chips, Doritos nacho cheese corn chips, and Smartfood cheese popcorn) or minimally processed (grapes, apple slices, clementine sections, grape tomatoes, unsalted peanuts, and baby carrots) snack foods, presented in counterbalanced order. Both sweet (desserts or fruit) and non-sweet (salty snacks or vegetables) food items were presented to account for differences in preferences. Participants were allotted 15 minutes to taste and rate a small amount of each food item, and told that they were welcome to consume any remaining food items (but that they would not be able to take any foods home). Energy intake (kcal) and percent (amount (g) consumed/ amount (g) offered) of test foods consumed were calculated by covert weighing of each individual food item before and after the taste test.

### Anthropometrics

Height was measured at the PEAS baseline assessment (< 12 weeks gestation) using a wall-mounted stadiometer to the nearest 0.1 cm, and weight was measured at each PEAS assessment using a standing scale to the nearest 0.1 kg; early pregnancy BMI was calculated from these measures. Last measured pregnancy weight was obtained from medical records (accessed January, 2018). GWG was calculated by subtracting early pregnancy weight from the last measured pregnancy weight (mean ± SD = 0.35 ± 0.75 weeks before delivery). We used measured early pregnancy weight rather than self-reported pre-pregnancy weight to calculate GWG because weight gain is minimal in the first trimester of pregnancy and since measured early pregnancy weight provides a less biased estimate of GWG [28]. The 2009 Institute of Medicine guidelines were used to classify excessive total GWG according to early pregnancy BMI and gestational age at delivery [29]. Three postpartum weight change variables were evaluated, including the percent of GWG lost at 6 months postpartum, the percent of GWG lost at 12 months postpartum, and a binary variable indicating whether the participant returned to early pregnancy weight at any of the postpartum visits.

### Self-reported eating behaviors

Between early pregnancy and 28 weeks’ gestation, PEAS participants completed the Dutch Eating Behavior Questionnaire (DEBQ), a 33-item validated measure of restrained eating (10 items), emotional eating (13 items), and external eating (10 items) [14]. Response options are on a 5-point Likert scale from 1 = never to 5 = very often. Subscale scores are calculated by averaging subscale item scores. Higher scores indicate more frequent behaviors in the corresponding domain. Internal consistency for the subscales in the full PEAS sample was α = 0.83 for restraint, α = 0.93 for emotional eating, and α = 0.83 for external eating.

### Depression

Participants completed the 10-item Edinburgh Postnatal Depression Scale [30] before 15 weeks’ gestation. Items ask about frequency of feelings over the preceding 7 days. Examples include: “I have been anxious or worried for no good reason”, and “I have felt sad or miserable”. Wording for the four-category response options vary across items; example response options include: 0 (*yes, most of the time*), 1 (*yes, quite often*), 2 (*not very often*), and 3 (*no, not at all*). Internal consistency in the full PEAS sample was α = 0.87 [31]. The sum of items determines the total score, ranging from 0 to 30, with higher scores indicating greater depressive symptoms.

### Stress

At at ≤ 28 weeks’ gestation, participants reported perceived stress over the preceding month, using the 10-item Perceived Stress Scale [32]. Items ask respondents how often they felt or thought certain ways, with 5-point Likert-type response options ranging from 0 (*never*) to 4 (*very often*). Examples include “how often have you been upset because of something that happened unexpectedly?”, “how often have you felt nervous and ‘stressed’?”, and “how often have you felt that things were going your way?”. Internal consistency in the full PEAS sample was α = 0.89 [31]. The sum of items determines the total score, ranging from 0 to 40, with higher scores indicating higher stress.

### Sleep quality

PEAS participants completed the 19-item Pittsburgh Sleep Quality Index [33] at ≤15 weeks gestation. Items assess multiple aspects of sleep duration and quality over the past month. Internal consistency in the full PEAS sample was α = 0.74 [31]. Total scores range from 0 to 21, with higher scores indicating lower sleep quality.

### Sociodemographic characteristics

Participants’ age and current gestational age were abstracted from the electronic medical record (accessed January, 2018) at the PEAS baseline assessment. Household income, household composition, and race/ethnicity were self-reported. Household income and composition were used to calculate the income-poverty ratio according to 2016 poverty thresholds [34]. The income-poverty ratio is a continuous measure that reflects household income relative to the poverty level corresponding to household composition (number of children and adults in the home), with higher values indicating higher income relative to poverty.

### Statistical analyses

Means ± SD and frequencies of sample characteristics were calculated. The temporality and distributions of the variables determined the analytic approach for estimating relationships with EAH. Regression models estimated relations of weight indicators, eating behaviors, sleep, stress, and depression with EAH. In models estimating associations with pregnancy weight-related eating behaviors and BMI, which preceded the EAH assessments, EAH intake served as the dependent variable; anthropometric variables measured after the EAH assessment were treated as dependent variables. Linear regression estimated associations of EAH with percent of GWG lost at 6 months and 12 months. GWG adequacy was dichotomized to reflect exceeding versus not exceeding the guidelines due to the very low frequency of inadequate GWG (n = 3, 7%). Logistic regression estimated relations with GWG adequacy (with not exceeding the guidelines as the referent category) and returning to early pregnancy weight (versus not). Three subjects were missing responses to the self-reported eating behaviors, depression, and sleep quality questionnaires; two subjects were missing responses to the stress questionnaire. Complete case analysis was used due to the low proportion of missingness. StataSE version 18 was used for all analyses, and *p* < .05 was used to determine statistical significance.

## Results

Mean ± SD early pregnancy age of the participants was 30.9 ± 3.4 years, 73% of participants reported non-Hispanic white race/ethnicity, approximately half of participants had early pregnancy BMI ≥ 25 kg/m^2^, and more than half of participants had excessive GWG (Table 1).

**Table 1:** Characteristics of the subsample of participants in the Pregnancy Eating Attributes Study who completed the Eating in the Absence of Hunger assessments (n = 46).

Characteristic	Distribution^1^
Age, years	30.9 ± 3.4
Early pregnancy BMI, kg/m^2^	
18.5 ≤ BMI < 25	25 (54)
25 ≤ BMI < 30	12 (26)
30 ≤ BMI	9 (20)
Gestational weight gain adequacy^3^	
Inadequate	3 (7)
Adequate	16 (36)
Excessive	26 (58)
Gestational age at Eating in the Absence of Hunger assessment, weeks	23.8 ± 5.4
Race/ethnicity	
Hispanic	5 (11)
Non-Hispanic Asian, Native American, Pacific Islander	3 (7)
Non-Hispanic black	2 (4)
Non-Hispanic other	2 (4)
Non-Hispanic white	33 (73)
Income-poverty ratio	4.0 ± 1.8
Edinburgh Postpartum Depression Scale score (5.1)	5.4 ± 2.8
Perceived Stress Scale score (13.08)	13.4 ± 6.1
Pittsburgh Sleep Quality Index score (6.07)	5.4 ± 4.1

^1^Values are mean ± SD or n (%)

^2^Measured in early pregnancy (< 12 weeks gestation)

^3^Categorized according to the 2009 Institute of Medicine guidelines [29].

**Fig 2:**
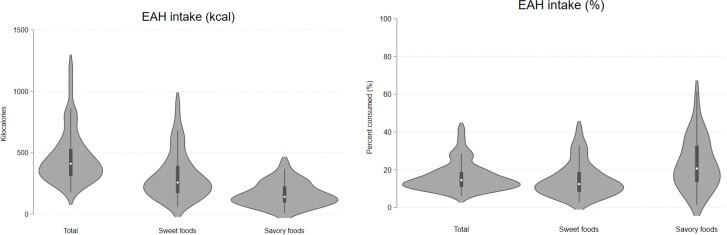
Eating in the absence of hunger (kcal and percent consumed) of foods overall and separately by type in adult participants in their 2^nd^ pregnancy trimester (n = 46). Sweet foods included cookies, brownies and Resse’s peanut butter cups; savory foods included Ruffles potato chips, Doritos nacho cheese corn chips, and Smartfood cheese popcorn. Mean (min – max) EAH kcal of all foods = 466 (176–1196), sweet = 311, (62–907), and savory = 161 (10 - 426). Mean (min – max) EAH % of all foods = 16 (6–42), sweet = 15 (3–42), savory = 23 (2–61).

As reported previously, mean ± SE EAH of highly processed foods was 461.3 ± 24.5 kcal (16.3% ± 1.1%) overall, 309.7 ± 21.0 kcal (14.7% ± 1.6%) for sweet foods, and 151.6 ± 51.4 kcal (21.9% ± 1.9%) for savory foods [13]. There was a large range of EAH energy intake (total range = 1020 kcal). The range of EAH energy intake from sweet foods (total range = 845 kcal) was larger than that of savory foods (total range = 416 kcal), while the range of percent intake from savory foods (total range = 59%) was larger than that of sweet foods (total range = 39%) (Fig 2).

Coefficient estimates in models examining relationships of eating behaviors with EAH of highly processed foods were mostly positive for emotional and external eating and negative for restrained eating (Table 2). However, standard errors were large, and none of the associations of eating behaviors with EAH reached statistical significance. Coefficients estimating associations of depressive symptoms, stress, and sleep quality with EAH were close to zero. Associations of self-reported eating behaviors and indicators of well-being with EAH of minimally processed foods were generally of smaller magnitude and the directions of associations were less consistent (S1 Table); there were no statistically significant relationships except for a positive association of sleep quality scores (indicating worse sleep quality) with EAH-kcal of savory food.

**Table 2:** Relationships^1^ of Eating in the Absence of Hunger (energy intake and percent of offered consumed) of highly-processed foods^2^ with eating behaviors, depressive symptoms, stress, and sleep quality during the 2^nd^ pregnancy trimester^3^.

	Energy intake, kcal						Percent intake					
	Total		Sweet		Savory		Total		Sweet		Savory	
Independent variable	β (SE)	*P*	β (SE)	*P*	β (SE)	*P*	β (SE)	*P*	β (SE)	*P*	β (SE)	*P*
BMI, kg/m^2^	5.1 (6.3)	0.43	3.5 (5.5)	0.53	2.2 (2.8)	0.44	0.2 (0.2)	0.43	0.2 (0.3)	0.50	0.3 (0.4)	0.50
Eating Behaviors												
Restrained	−57.4 (60.2)	0.35	−25.6 (52.9)	0.63	−25.3 (26.1)	0.34	−1.9 (2.1)	0.38	−1.1 (2.5)	0.66	−3.5 (3.7)	0.35
Emotional	23.5 (47.8)	0.63	−19.7 (41.1)	0.63	33.5 (20.1)	0.10	0.9 (1.7)	0.60	−0.8 (1.9)	0.70	4.9 (2.9)	0.09
External	91.6 (77.9)	0.25	43.4 (68.9)	0.53	45.1 (33.6)	0.19	3.3 (2.7)	0.24	2.2 (3.3)	0.51	6.7 (4.8)	0.17
Depressive symptoms	−11.6 (7.9)	0.15	−6.7 (7.0)	0.35	−5.4 (3.5)	0.13	−0.4 (0.3)	0.18	−0.3 (0.3)	0.37	−0.8 (0.5)	0.14
Stress	0.6 (5.7)	0.91	3.3 (5.0)	0.52	−2.9 (2.4)	0.25	0.04 (0.2)	0.83	0.2 (0.2)	0.49	−0.4 (0.3)	0.25
Sleep quality	10.7 (11.8)	0.37	13.8 (10.2)	0.18	−1.3 (5.3)	0.81	0.4 (0.4)	0.31	0.7 (0.5)	0.16	−0.1 (0.7)	0.85

^1^Estimates from linear regression models predicting EAH (kcal and percent consumed) of all foods and separately for sweet and savory foods.

^2^Sweet foods included chocolate chip cookies, brownies, Reese’s peanut butter cups; savory foods included Ruffles potato chips, Doritos nacho cheese corn chips, and Smartfood cheese popcorn.

^3^Eating behaviors, depressive symptoms, stress, and sleep quality were assessed using validated questionnaires.

Relationships of EAH with pregnancy-related weight change variables were mostly null (Table 3). Odds ratios estimating associations of EAH with odds of excessive gestational weight gain were within a very small margin of 1.00; confidence intervals were also small, and all included the null association. Point estimates for regression coefficients predicting postpartum weight change from EAH were all negative, suggesting an inverse association, but only the inverse association of savory highly-processed foods (both energy intake and percent intake) with percent of GWG lost at 6 months postpartum was statistically significant. Similarly, there were no statistically significant associations of EAH of minimally processed foods with pregnancy weight change variables (S2 Table).

**Table 3:** Relationships of EAH with GWG and postpartum weight change.

	Excessive GWG^1^		Ever returned to early pregnancy weight^2^		% of GWG lost – 6 months postpartum^3^		% of GWG lost – 12 months postpartum	
Independent variable	OR (95%CI)^1^	*P*	OR (95%CI)^1^	*P*	β (SE)^2^	*P*	β (SE)^2^	*P*
EAH – kcal								
Total	1.00 (1.00–1.00)	0.44	1.00 (1.00–1.00)	0.41	−0.02 (0.02)	0.30	−0.01 (0.03)	0.70
Sweet	1.00 (1.00–1.00)	0.45	1.00 (1.00–1.00)	0.34	−0.006 (0.03)	0.82	−0.004 (0.03)	0.89
Savory	1.00 (0.99–1.01)	0.78	1.00 (0.99–1.01)	0.61	−0.1 (0.6)	0.03	−0.07 (0.07)	0.32
EAH – percent								
Total	1.04 (0.95–1.13)	0.42	0.97 (0.89–1.05)	0.44	−0.59 (0.67)	0.38	−0.23 (0.80)	0.78
Sweet	1.03 (0.96–1.10)	0.44	0.97 (0.91–1.04)	0.35	−0.09 (0.56)	0.87	−0.08 (0.67)	0.91
Savory	0.99 (0.95–1.04)	0.74	0.99 (0.94–1.04)	0.67	−0.83 (0.39)	0.04	−0.45 (0.51)	0.38

EAH – eating in the absence of hunger, GWG – gestational weight gain

^1^Outcome defined as exceeding guidelines for total pregnancy weight gain versus gaining within the 2009 Institute of Medicine guidelines (33).

^2^Outcome defined as whether participant returned to early pregnancy weight or below at 6 weeks, 6 months, or 12 months postpartum.

^3^Outcome defined as the percent of total GWG lost at 6 months or 12 months postpartum.

## Discussion

In this study, early pregnancy BMI, self-reported eating behaviors, and well-being indicators were not associated with EAH of highly-processed foods during pregnancy, and EAH was not strongly or consistently associated with pregnancy or postpartum weight change. Based on the established conceptual frameworks for these constructs, early pregnancy BMI, emotional and external eating, depressive symptoms, stress, and poor sleep quality were hypothesized to predict greater EAH, while there was no prespecified hypothesis regarding the direction of the relationship of restrained eating with EAH. Similarly, since EAH is considered a risk factor for eating beyond satiation leading to positive energy balance, it was hypothesized to be related to greater pregnancy and postpartum weight gain. Collectively, findings contradicted the hypothesized relationships.

This study adds to the sparse evidence on the relationship of EAH with prospective weight change. In a study of non-Hispanic white girls, EAH at age 5 years was positively associated with higher BMI z-score at age 9 years only in children of mothers with overweight at baseline [10]. However, EAH was not associated with weight gain over one year in two other prospective studies in children and adolescents [9,11]. Similarly, in college-aged females, a positive relationship of EAH with weight change over two months dissipated at follow-up assessments at 4-, 6-, and 12-months [7]. In the present study, EAH in the second pregnancy trimester was not related to baseline BMI, excessive gestational weight gain, or postpartum weight change. Thus, weight status did not predict EAH, and EAH did not predict excessive weight gain or weight retention. In contrast, we previously demonstrated that EAH of highly processed foods, particularly sweet foods, was related to worse diet quality assessed throughout pregnancy [12]. Taken together, these findings suggest that while EAH during pregnancy may be generalizable to typical diet quality, it may not be a useful model for typical contexts that lead to positive energy balance in naturalistic settings. In post hoc analyses, factors were similarly unrelated to intake during the standardized meal, suggesting that, during pregnancy, meal and post-meal intake at two occasions in controlled settings may have limited generalizability to typical contexts for excessive energy intake. EAH assessed during pregnancy may differ from when not pregnant, given research suggesting that pregnancy acts as a “disinhibitor” for eating behavior (i.e., decreases motivation to curtail excessive intake) [18], which could lead to differential associations with weight indicators as compared with during other developmental periods. Additional studies are needed to confirm these results and test whether EAH differs in pregnancy, given the limited available evidence.

The null relationships of self-reported eating behaviors with EAH are consistent with previous research in non-pregnant samples [7,15–17], although contrary to hypotheses. Restrained, external, and emotional eating are each conceptualized as factors contributing to overeating. External eating, the tendency to overeat in response to food stimuli including the presence of foods [14], was hypothesized to be positively associated with EAH. In one study, emotional eating assessed by the TFEQ was unrelated to EAH, while in another, emotional eating was related to greater EAH only after a manipulation of affect [17]; the absence of an affective state manipulation in the present study may explain the null association observed herein. Associations of restrained eating, the tendency to eat less than desired for the purpose of weight control, with energy intake in laboratory settings are inconsistent [15–17,35]. While evidence suggests that dietary restraint decreases during pregnancy versus pre-pregnancy [18], it is unknown whether emotional or external eating differ during pregnancy. The available evidence suggests that these eating behaviors do not strongly predict variability in EAH during pregnancy. Additionally, if EAH during pregnancy is not generalizable to typical contexts that lead to excessive energy intake throughout pregnancy, then this could have contributed to the null associations with the eating behaviors examined. Future research testing whether emotional, external, or restrained eating differ during pregnancy or are differentially related to food intake during pregnancy versus other periods would be informative.

The mean depression, stress, and sleep quality scores in this sample were similar, but slightly more severe, than those reported in larger samples of pregnant participants [36–38], which could have led to stronger relationships with EAH. Despite this, findings from this study indicate that sleep, stress, and depression did not explain variability in EAH during pregnancy. Although some evidence suggests that higher depression, stress, and worse sleep quality were associated with higher body weight and greater weight change in non-pregnant samples [39–43], experimental studies investigating relationships with food intake are few, and their findings are inconsistent. Previous studies (in non-pregnant samples) have demonstrated both inverse and null associations of sleep [44–47] and stress [24,35,48] with EAH. While the relationship of depression with EAH has not been examined, one study found a positive association of depressive symptoms with energy intake at a buffet meal, although this was not observed in all subgroups [49]. Some evidence suggests that dietary restraint [49], disinhibition [24], and emotional eating [35] may modify relationships of sleep, stress, and depression with EAH. Collectively, the available evidence suggests that these relationships are complex and likely to depend on a variety of individual and contextual factors.

Strengths of this study include the assessment of EAH under controlled conditions, which enables precise intake measurement and is not prone to response bias; however, eating in a laboratory may lead to reactivity bias, which could limit generalizability to typical intake in naturalistic settings. Additionally, eating behaviors, stress, sleep, and depression were measured using validated measures, although all of these self-report measures are susceptible to reporting bias. While the small sample size could have limited statistical power for detecting significant associations of self-reported eating behaviors with EAH due to the wide confidence intervals, the point estimates for associations of EAH with weight outcomes were close to zero and the confidence intervals were very narrow, indicating the magnitudes of any true underlying relationships may be quite small and of limited clinical significance.

## Conclusion

In this prospective study, EAH during pregnancy was not associated with early pregnancy BMI or weight change during pregnancy or postpartum. Additionally, there was no evidence of associations of EAH during pregnancy with restrained, emotional, or external eating, nor with depression, stress, or sleep quality. Future research investigating prospective associations of EAH with weight change and behavioral risk factors conducted in larger samples and in participants of different developmental periods could help determine the utility of laboratory EAH measurement as an indicator of behavioral susceptibility to excessive energy intake leading to weight gain. Further refinement of the EAH paradigm to improve its resemblance to instances of overeating in naturalistic settings may help improve its generalizability.

## Supporting information

S1 Table Relationships^1^ of Eating in the Absence of Hunger (energy intake and percent of offered consumed) of minimally-processed foods^2^ with eating behaviors, depressive symptoms, stress, and sleep quality during the 2^nd^ pregnancy trimester^3^. ^1^Estimates from linear regression models predicting eating in the absence of hunger of all foods and separately for sweet and savory foods. ^2^Sweet foods included grapes, apples, and clementines; non-sweet foods included grape tomatoes, peanuts, and baby carrots. ^3^Eating behaviors, depressive symptoms, stress, and sleep quality were assessed using validated questionnaires.
(DOCX)

S2 Table Relationships of EAH of minimally processed foods with gestational weight gain (GWG) and postpartum weight change. EAH – eating in the absence of hunger, GWG – gestational weight gain. ^1^Outcome defined as exceeding guidelines for total pregnancy weight gain versus gaining within the 2009 Institute of Medicine guidelines (33). ^2^Outcome defined as whether participant returned to early pregnancy weight or below at 6 weeks, 6 months, or 12 months postpartum. ^3^Outcome defined as the precentage of total gestational weight gain lost at 6 months or 12 months postpartum.
(DOCX)

S1 Data De-identified data and variable labels to enable replication of the analyses described in this paper. (XLSX)
