# Supplementary material for: Eating in the absence of hunger is not associated with weight, self-reported eating behaviors, or well-being in pregnant adults: Prospective cohort study
Source: PLoS One. 2025 Jun 24;20(6):e0325478. doi: 10.1371/journal.pone.0325478 (PMC12186914; doi:10.1371/journal.pone.0325478)
Supplement: S1 Table — 1Estimates from linear regression models predicting eating in the absence of hunger of all foods and separately for sweet and savory foods. 2Sweet foods included grapes, apples, and clementines; non-sweet foods included grape tomatoes, peanuts, and baby carrots. 3Eating behaviors, depressive symptoms, stress, and sleep quality were assessed using validated questionnaires. (DOCX) [file pone.0325478.s001.docx]

**S1 Table.** Relationships^1^ of Eating in the Absence of Hunger (energy intake and percent of offered consumed) of minimally-processed foods^2^ with eating behaviors, depressive symptoms, stress, and sleep quality during the 2^nd^ pregnancy trimester^3^.

|  | **Energy intake, kcal** | | | | | | **Percent intake** | | | | | |
| --- | --- | --- | --- | --- | --- | --- | --- | --- | --- | --- | --- | --- |
|  | **Total** | | **Sweet** | | **Savory** | | **Total** | | **Sweet** | | **Savory** | |
| **Independent variable** | **β (SE)** | ***P*** | **β (SE)** | ***P*** | **β (SE)** | ***P*** | **β (SE)** | ***P*** | **β (SE)** | ***P*** | **β (SE)** | ***P*** |
| BMI, kg/m^2^ | 0.6 (2.0) | 0.78 | 0.4 (1.1) | 0.73 | 0.2 (1.5) | 0.91 | -0.01 (0.2) | 0.96 | 0.2 (0.4) | 0.6 | -0.4 (0.3) | 0.25 |
| Eating Behaviors |  |  |  |  |  |  |  |  |  |  |  |  |
| Restrained | 5.1 (18.6) | 0.79 | -8.7 (10.0) | 0.39 | 13.9 (13.9) | 0.33 | -2.4 (2.1) | 0.26 | -3.5 (3.4) | 0.32 | -1.7 (2.9) | 0.56 |
| Emotional | 26.0 (15.3) | 0.10 | 10.9 (8.4) | 0.20 | 15.1 (11.7) | 0.20 | 2.3 (1.8) | 0.21 | 3.5 (2.9) | 0.24 | 1.5 (2.5) | 0.53 |
| External | 22.2 (25.2) | 0.38 | 5.8 (13.7) | 0.68 | 16.4 (19.0) | 0.39 | 2.4 (2.9) | 0.41 | 1.7 (4.7) | 0.73 | 5.1 (3.9) | 0.20 |
| Depressive symptoms | 0.81 (2.6) | 0.76 | -0.72 (1.4) | 0.62 | 1.5 (1.9) | 0.43 | -0.06 (0.3) | 0.84 | -0.1 (0.5) | 0.80 | 0.001 (0.4) | 0.997 |
| Stress | 0.54 (1.8) | 0.77 | -0.46 (0.99) | 0.65 | 1.0 (1.4) | 0.47 | -0.1 (0.2) | 0.54 | -0.2 (0.3) | 0.65 | -0.2 (0.3) | 0.59 |
| Sleep quality | 3.5 (3.7) | 0.35 | -3.6 (2.0) | 0.08 | 7.0 (2.6) | 0.009 | -0.6 (0.4) | 0.14 | -1.3 (0.7) | 0.06 | 0.3 (0.6) | 0.64 |

^1^Estimates from linear regression models predicting eating in the absence of hunger of all foods and separately for sweet and savory foods.

^2^Sweet foods included grapes, apples, and clementines; non-sweet foods included grape tomatoes, peanuts, and baby carrots.

^3^Eating behaviors, depressive symptoms, stress, and sleep quality were assessed using validated questionnaires.
