# Supplementary material for: Eating in the absence of hunger is not associated with weight, self-reported eating behaviors, or well-being in pregnant adults: Prospective cohort study
Source: PLoS One. 2025 Jun 24;20(6):e0325478. doi: 10.1371/journal.pone.0325478 (PMC12186914; doi:10.1371/journal.pone.0325478)
Supplement: S2 Table — EAH – eating in the absence of hunger, GWG – gestational weight gain. 1Outcome defined as exceeding guidelines for total pregnancy weight gain versus gaining within the 2009 Institute of Medicine guidelines (33). 2Outcome defined as whether participant returned to early pregnancy weight or below at 6 weeks, 6 months, or 12 months postpartum. 3Outcome defined as the precentage of total gestational weight gain lost at 6 months or 12 months postpartum. (DOCX) [file pone.0325478.s002.docx]

**S2 Table.** Relationships of EAH of minimally processed foods with gestational weight gain (GWG) and postpartum weight change.

|  | **Excessive GWG^1^** | | **Ever returned to**  **early pregnancy weight^2^** | | **% of GWG lost –**  **6 months postpartum^3^** | | **% of GWG lost –**  **12 months postpartum** | |
| --- | --- | --- | --- | --- | --- | --- | --- | --- |
| **Independent variable** | **OR (95%CI)^1^** | ***P*** | **OR (95%CI)^1^** | ***P*** | **β (SE)^2^** | ***P*** | **β (SE)^2^** | ***P*** |
| EAH – kcal |  |  |  |  |  |  |  |  |
| Total | 1.00 (0.99 – 1.01) | 0.99 | 0.99 (0.98 – 1.00) | 0.06 | -0.07 (0.07) | 0.33 | -0.1 (0.09) | 0.25 |
| Sweet | 1.00 (0.98 – 1.02) | 0.93 | 0.98 (0.97 – 1.00) | 0.08 | -0.16 (0.1) | 0.25 | -0.2 (0.16) | 0.20 |
| Savory | 1.00 (0.99 – 1.01) | 0.97 | 0.99 (0.98 – 1.00) | 0.22 | -0.05 (0.09) | 0.65 | -0.07 (0.12) | 0.55 |
| EAH – percent |  |  |  |  |  |  |  |  |
| Total | 0.99 (0.92 – 1.07) | 0.92 | 0.92 (0.84 – 1.01) | 0.07 | -0.7 (0.6) | 0.28 | -0.9 (0.8) | 0.24 |
| Sweet | 1.00 (0.96 – 1.05) | 0.91 | 0.95 (0.90 – 1.01) | 0.09 | -0.5 (0.4) | 0.25 | -0.7 (0.5) | 0.16 |
| Savory | 0.97 (0.92 – 1.03) | 0.39 | 0.98 (0.92 – 1.04) | 0.49 | -0.04 (0.5) | 0.93 | 0.1 (0.6) | 0.81 |

EAH – eating in the absence of hunger, GWG – gestational weight gain

^1^Outcome defined as exceeding guidelines for total pregnancy weight gain versus gaining within the 2009 Institute of Medicine guidelines (33).
^2^Outcome defined as whether participant returned to early pregnancy weight or below at 6 weeks, 6 months, or 12 months postpartum.

^3^Outcome defined as the precentage of total gestational weight gain lost at 6 months or 12 months postpartum.
